# Papillary Tumor of the Pineal Region Rare Pediatric CNS Tumor Case Series Treated in King Fahad Medical City (KFMC)

**DOI:** 10.3390/curroncol29100595

**Published:** 2022-10-10

**Authors:** Nahla A. Mobark, Musa Alharbi, Fahad Alotabi, Azhar Alshoumer, Wafa Al Shakweer, Zaid G. AlNaqib, Abdulaziz N. AlSaad, Ali O. Balbaid, Ebtehal Alsolme, Malak S. Abedalthagafi

**Affiliations:** 1 Department of Pediatric Oncology Comprehensive Cancer Centre, King Fahad Medical City, Riyadh 12231, Saudi Arabia; 2 Pediatric Neurosurgical Department, King Fahad Medical City, Riyadh 12231, Saudi Arabia; 3 Department of Pathology & Laboratory Medicine, King Fahad Medical City, Riyadh 12231, Saudi Arabia; 4 Radiology Department, King Fahad Medical City, Riyadh 12231, Saudi Arabia; 5 Radiation Oncology Department, Comprehensive Cancer Centre, King Fahad Medical City, Riyadh 12231, Saudi Arabia; 6 Genomics Research Department, King Fahad Medical City, Riyadh 12231, Saudi Arabia; 7 Department of Pathology & Laboratory Medicine, Emory University, Atlanta, GA 30322, USA

**Keywords:** PTPR, pineal tumor, pediatric tumor, copy number variants, methylation profile

## Abstract

The clinical behaviors, prognosis, and appropriate treatments of papillary tumors of the pineal region (PTPR) are not fully defined due to the rarity of these tumors. At diagnosis, PTPR may present with clinical symptoms, including headache with obstructive hydrocephalus, diplopia, vomiting, and lethargy, as well as neurological signs, including Argyll Robertson pupils and Parinaud’s syndrome due to compression of the dorsal midbrain, specifically the periaqueductal region with horizontal nystagmus. Radiological assessment of pineal region lesions is challenging, with a wide range of potential differential diagnoses. PTPR typically presents as a heterogeneous, well-circumscribed mass in the pineal region, which might contain cystic areas, calcifications, hemorrhages, or protein accumulations. Here, we report three female pediatric patients with PTPR treated in King Fahad Medical City (KFMC) in Saudi Arabia. Histological and immunohistochemical diagnosis was confirmed by analysis of genome-wide DNA methylation profiles. This case series expands on the available reports on the clinical presentations of PTPR and provides important information on the responses to different treatment modalities.

## 1. Introduction

Papillary tumors of the pineal region (PTPR) are very rare neuroepithelial tumors characterized by a papillary architecture and epithelial cytology. First introduced in the World Health Organization classification of central nervous system tumors in 2007 and classified as WHO grade 2–3 [1,2].

PTPR has no sex predilection, although some reports suggested a female preponderance. Fewer than 200 cases are reported to date, and pediatric cases are very rare and have an average patient age of 11.6 years (range 1–18 years) [3]^.^

PTPR has an uncertain origin; it does not arise from the pineal gland but is thought to arise from the ependymal cells of the subcommissural organ (SCO), which is located below the posterior commissure at the level of the cerebral aqueduct, just anterior to the pineal gland. The SCO contains specialized cytokeratin-positive and nestin-positive ependymal cells and is involved in the secretion of glycopeptides and regulation of CSF during development. Its glycopeptide content is thought to be the source of the intrinsic T1 hyperintensity commonly reported in PTPR [4]^.^

DNA methylation-based tumor classification emerged as a promising tool for CNS tumors. Two methylation groups for papillary tumors of the pineal region (A and B) are defined. In “PTPR Group A”, which is equivalent to PTPR Group 1 in the report of Heim et al. [5], loss of chromosome 10 was reported. Chromosome 10 loss in PTPR is linked to PTEN mutations and activation of the phosphoinositide 3-kinase/Akt/mTOR signaling pathway [6]. Other molecular alterations currently remain unclear. Numeric whole chromosome changes are frequently observed in this class, with gain in chromosomes 4, 5, 7, 11, 12, 16, and 18, and loss of chromosomes 1 and 10 in over 50% of cases. “PTPR Group B” is equivalent to PTPR Group 2 in the report of Heim et al. [5] and shows a characteristic copy number variation (CNV) profile. Numeric whole chromosome changes are frequent in this class, with gain in chromosome 8 (>60%) and loss of Chr. 3 (>50%) and Chr. 10 (100%). A more aggressive clinical course is recommended for “PTPR Group B” [5].

Here, we report a case series involving three PTPR pediatric patients treated in King Fahad Medical City. All were female, and the histological and immunohistochemical diagnosis was confirmed by methylation CNS classifier profiling. Samples were processed in our institutional genomics facility, which combined samples from multiple sources for processing by an Illumina Infinium HumanMethylation450 BeadChip (450k) array or by a MethylationEPIC BeadChip (850k) array. Standard quality controls confirmed adequate tumor purity/quality, bisulfite conversion, and DNA quality. IDAT files were uploaded to either version 11b2 or 11b4 of the online CNS tumor methylation classifier (https://www.molecularneuropathology.org), (accessed on 1 May 2021), and reports were produced as shown by Capper et al. [7]^.^

## 2. Case #1

**Figure 1:**
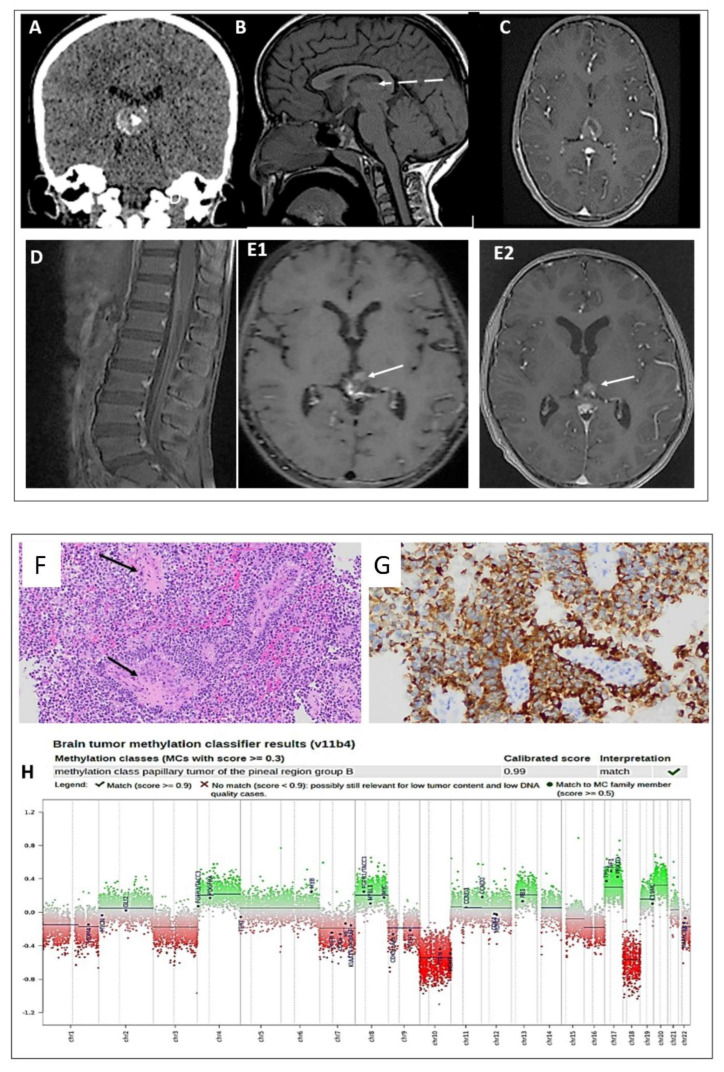
(**A**) CT scan shows a hyperdense lesion with a focus of central calcification. (**B**) SE T1W MRI shows an iso-intense lesion with a small peripheral area of hyperintensity (dashed arrow). (**C**) Postcontrast T1W MRI shows heterogeneous enhancement of the lesion. (**D**) Sagittal postcontrast T1W MRI shows leptomeningeal thickening and enhancement along the surfaces of the conus medullaris and cauda equina nerve roots (**E1**,**E2**). Postcontrast T1W MRI performed four months and four years later shows a stable, residual enhanced focus (arrow) with no sign of recurrence. Histologic features of the primary tumor assessed by routine hematoxylin and eosin staining demonstrating the following: (**F**) a pseudopapillary and solid growth pattern (arrows), (**G**) strongly positive CK18 immunostaining, (**H**) DNA methylation class PTPR Group B, and a copy number plot showing loss of Chr. 10.

A 9-year-old girl presented to the local hospital with headache and vomiting after mild head trauma and cervical lymphadenopathy. A CT scan identified hydrocephalus and a ventriculoperitoneal shunt (VPS) was placed. The patient was transferred to KFMC for further evaluation. A brain CT and MRI identified a pineal region mass of 1.6 × 1.9 × 2 cm (Figure 1A–C). MRI scans of the spine showed leptomeningeal thickening along the conus medullaris (Figure 1D), suggesting CSF spinal seeding metastasis.

The patient underwent an endoscopic radiotherapy frontal stereotactic biopsy of the pineal tumor only. Histopathology revealed a grade 2–3 papillary tumor of the pineal region (PTPR) (Figure 1F,G). Methylation classification analysis revealed a papillary tumor of the pineal region group B (Figure 1H). CSF cytology was negative for malignant cells.

Due to the MRI findings suggestive of spinal seeding metastasis, the patient underwent craniospinal irradiation treatment. A follow-up MRI 4 months after radiation intracranial pressure revealed a significant decrease in the size of the heterogeneous residual lesion along the surgical bed (Figure 1(E1)). No evidence of spinal CSF seeding metastasis was detected.

Four years post-diagnosis, the patient remains in complete remission, with no symptoms or signs of tumor recurrence (Figure 1(E2)).

## 3. Case #2

An 8-year-old girl presented to the pediatric neurosurgery department at KFMC with a progressive history of headaches, obstructive hydrocephalus and decreased visual acuity. Neurological examination was positive for mild proptosis and Parinaud’s syndrome, with paralysis of the upward conjugate gaze, pupils nonreactive to light, but preserved accommodation reflex. Other cranial nerves were intact, and the patient had normal tone and strength of upper and lower limbs.

**Figure 2:**
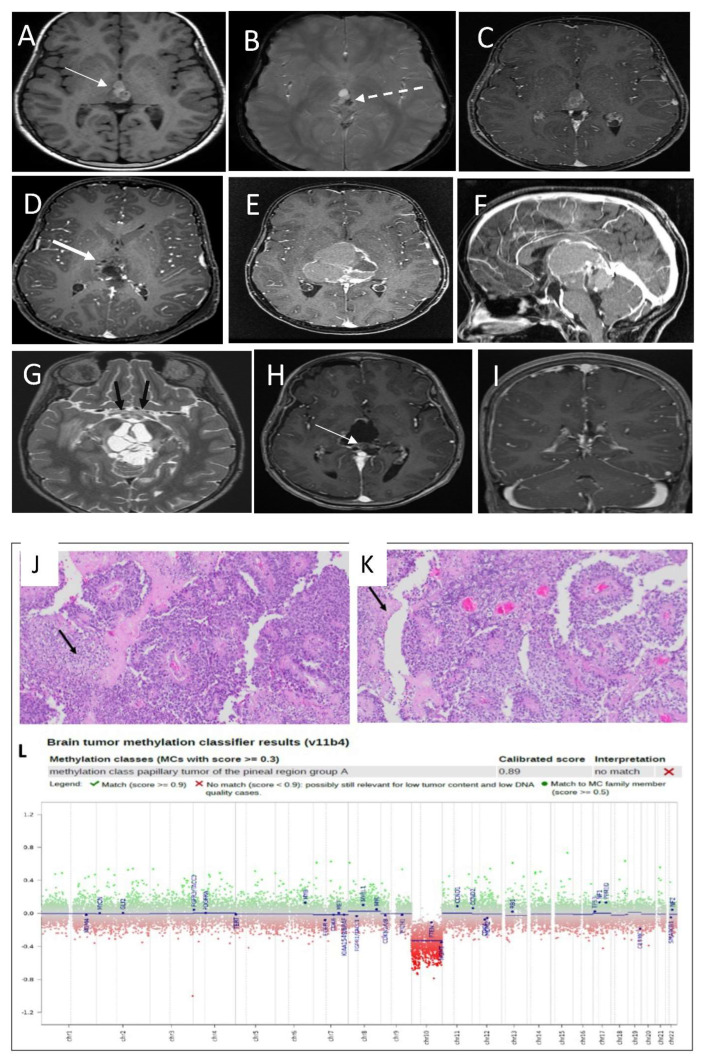
(**A**) Non-enhanced T1 SE WI shows a complex lesion with multiple areas of hyper-intensity within the lesion (thin white arrow). (**B**) MRI susceptibility weighted image shows areas of the susceptibility effect in the left side and posterior indicating a hemorrhage (dashed arrow) but the area in the anterior aspect of the lesion is not corresponding to hemorrhage. (**C**) MRI T1 post contrast shows non-significant enhancement of the lesion. (**D**) Partial resection of the pineal region lesion with a residual lesion (thick arrow). (**E**) MRI T1 post contrast 3 months post first surgery showed a significant disease recurrence with more lesion morphological changes. (**F**) MRI T1 post contrast in sagittal plans shows the mass effects in the adjacent structures. (**G**) T2 SE WI thin slice shows the stretching of the optic chiasm with abnormal signal (black arrow). (**H**) MRI post third surgical resection confirmed residual small residual tumor (arrow). (**I**) MRI follow-up post end of chemotherapy shows improvement of the surgical cavity and the residual lesion with no recurrence. Histologic features of the primary tumor assessed by routine hematoxylin and eosin staining demonstrate (**J**) a pseudopapillary growth pattern, with the solid area exhibiting clear vacuolated cytoplasm (arrow) and (**K**) a pseudopapillary growth pattern with areas of necrosis (arrow). (**L**) DNA methylation class PTPR group A.

A brain MRI showed pineal lesions (Figure 2A–C), and the spinal MRI was unremarkable (not shown). The patient underwent left craniotomy using the interhemispheric approach for an open biopsy of the pineal tumor and VP shunt insertion via a right frontal approach. A postoperative MRI showed residual tumor tissue in the pineal region, and in the third ventricle (Figure 2D).

After surgery, the patient recovered well and was ambulating independently, and she was discharged home with plans for observation and MRI follow-up.

Histopathological analysis revealed a grade 2–3 papillary tumor of the pineal region (PTPR) (Figure 2J,K). The tissue showed extensive necrosis, moderate proliferative index, and no mitotic figures. Methylation class analysis was a papillary tumor of the pineal region group A (Figure 2L).

She remained in good health until 4 months, when she was admitted to the neurosurgical ward with increased somnolence and decreased activity. MRI showed a progressive increase in the size of the residual tumor in the pineal region, now engulfing the tectal plate and obstructing the cerebral aqueduct (Figure 2E,F). Additionally, the MRI showed multiple areas of intratumoral bleeding, as well as calcification. This progression is associated with more hemorrhage and cystic changes, as well as greater T1 hyperintensity. The optic chiasm appeared to be relatively stretched, with abnormal T2 hyperintensity noted bilaterally along the optic tracts (Figure 2G). Spinal MRI was unremarkable (not shown).

She underwent a second craniotomy with partial resection and debulking of the progressed tumor. Postoperative results reveal a small residual lesion (Figure 2H).

Again, histopathological examination confirmed PTPR with a higher mitotic rate (over 15/10 hpf) accompanied by foci of necrosis, raising the possibility of a high-grade transformation (not shown). CSF analysis was negative for metastases. The patient received external beam radiation therapy (EBRT) localized to the brain at 59.4 Gy/33 Fx.

She recovered well, with no neurological deficits and was discharged in a good condition. Due to the aggressive pineloblastoma-like behavior of the tumor and recurrent relapse, she was initiated on the chemotherapy protocol of Medulloblastoma Saudi Arabian Pediatric Hematology Oncology Society (SAPHOS), with a total of six maintenance cycles alternating (A&B) as follows:

Cycle A: Cisplatin 90 mg/m2/day, day 1 and oral etoposide 35 mg/m2/day P.O. days 1–21 of a 4-week cycle.

Cycle B: Cyclophosphamide 1 g/m2/day, days 1 and 2, vincristine 1.5 mg/m2, days 1, 8, of a 4-week cycle with G-CSF SQ at 5 μg/kg/day, daily for at least 10 days, starting 24 hours after the last dose of chemotherapy in each cycle.

The patient tolerated chemotherapy well. More than one year post chemotherapy, she is clinically well, and her last MRI showed improvement of the postoperative changes, with no definite enhanced residual or recurrent masses at the surgical bed, and no intracranial or intraspinal CSF seeding metastasis (Figure 2I).

## 4. Case #3

A 6-year-old girl presented with a 3-month history of headache and vomiting. A CT scan conducted at a local hospital indicated a pineal tumor with obstructive hydrocephalus. She underwent surgical endoscopic ventriculostomy with EVD insertion, which was complicated by postoperative meningitis and was treated with a full course of antibiotics. Thereafter, a permanent VPS was inserted and the case was referred to KFMC for proper surgical management.

**Figure 3:**
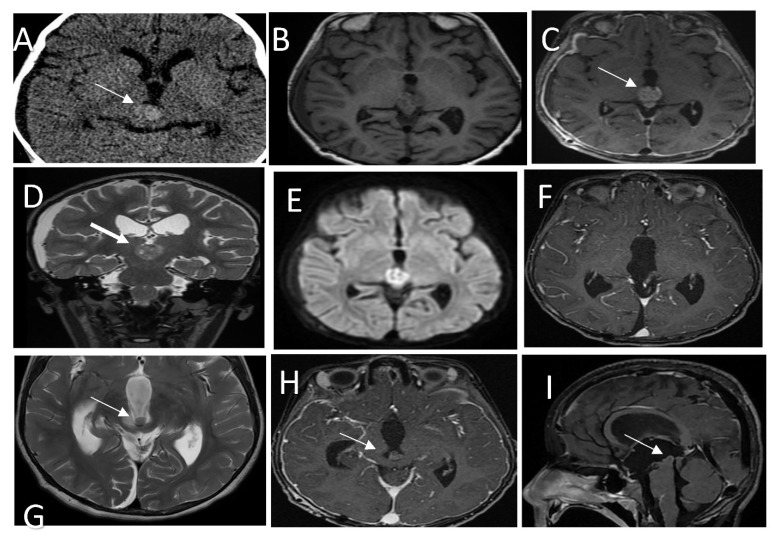

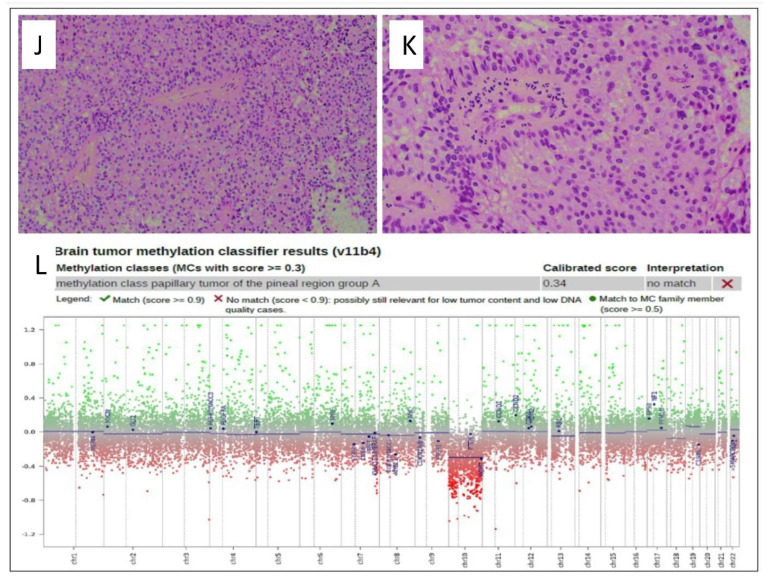
(**A**) CT scan shows a hyperdense pineal gland mass with no calcification (arrow). (**B**) SE T1W MRI shows an isointense lesion with peripheral areas of hyperintensity. (**C**) Post-contrast T1W MRI shows enhancement of the lesion (arrow). (**D**) T2WI in coronal plan shows a low signal intensity behavioral appearance of the lesion. (**E**) Diffusion weighted images show bright signal indication diffusion restriction (ADC not shown) (**F**) Post-contrast T1W MRI after the first resection shows gross total resection. (**G**) Follow-up MRI T2 WI shows a recurrent lesion in the posterior inferior aspect of the third ventricle (arrow) with mass effect. (**H**,**I**) Post contrast T1W MRI in axial and sagittal shows the recurrent lesion. Histologic features of the tumor assessed by routine hematoxylin and eosin staining demonstrate (**J**) a mainly solid growth pattern with round-oval nuclei and abundant clear cytoplasm and (**K**) short columnar perivascular cells with clear cytoplasm. (**L**) DNA methylation class PTPR Group A.

A pre surgery MRI showed a well-circumscribed, enhanced mass measuring 1.5 × 1.4 × 1.4 cm in the AP, CC, and transverse diameters, respectively, showing moderate diffusion restriction in the pineal region and extending into the inferior and posterior aspects of the third ventricle (Figure 3A–E). Spinal MRI was unremarkable (not shown). The patient underwent occipital craniotomy with a gross total surgical resection of the pineal mass (Figure 3F).

Histopathology analysis revealed a PTPR (Figure 3J,K) with papillary infiltrates showing necrotic foci and a moderate proliferative index and absence of mitotic activity.

Methylation class analysis identified a papillary tumor of the pineal region group B (Figure 3L). CSF cytology showed inflammatory cells. No malignant cells were detected (not shown).

As discussed in the multidisciplinary neuro-oncology tumor board, the patient was planned for observation and MRI follow-up. She remained well until one-year post-diagnosis, when she came to the ER with headache, lethargy, and signs of raised intracranial pressure (ICP), and was admitted to the PICU.

A brain MRI showed a recurrent pineal region tumor measuring 0.9 × 0.9 × 0.7 cm^3^ in AP, TR, and CC, respectively. The lesion was situated in the posterior aspect of the third ventricle and embedded in the proximal cerebral aqueduct, causing secondary obstructive hydrocephalus with a mass effect on the tectum, which was displaced posteriorly (Figure 3G–I); MRI of the spine was unremarkable (not shown).

She had emergency EVD insertion to relieve ICP. The neurosurgical team felt that the recurrent tumor was unresectable, so permanent right parietal VPS was inserted with marked clinical and neurological improvement. Lumbar puncture for CSF cytology showed atypical cells, indicating microscopic CSF dissemination. She was started on adjuvant CSI radiotherapy at 54 gray/30 fractions (CSI 36 gray/20 fractions + pineal boost 18 gray/10 fractions). She is currently stable, and MRI follow-up showed a stable residual mass in the pineal region (not shown).

## 5. Discussion

In this report, we describe a case series of three female pediatric patients, each with distinct clinical behaviors and treated in different ways, reflecting the heterogenicity of the tumor and lack of a consensus on proper therapeutic and management plans.

The radiological findings of PTPR in our case series mostly match those reported in the literature. However, our cases showed a heterogeneous signal intensity on T2WI with diffusion restriction and heterogeneous enhancement.

Although case #2 showed multiple large areas of high signal intensity on T1WI, other cases mostly presented with isosignal intensity on T1WI, with hyperintensity observed along the periphery of the lesion. Most of the high T1 signal intensity in our cases is attributed to hemorrhage, as the high signal on T1WI was decreased in the follow-up studies with a greater susceptibility effect, but other areas remained unchanged, and did not show any susceptibility and were not suppressed in fat saturated sequences, indicating proteinaceous content.

In a study by Chang et al. [8], the authors reported a high T1WI signal intensity and intrinsic T1 hyperintensity within the PTPR lesion of an adult patient after confirming the absence of fat, hemorrhage, melanin, or calcification in a mass of the posterior commissure or pineal region [8].

Similarly, a case report study on a 17-year-old patient found a T1 hyperintensity within the lesion after the authors excluded fat content and calcification. The authors concluded that the glycopeptides secreted from PTPR were likely the cause for this T1 hyperintensity [9].

Interestingly, images in our young pediatric patients showed a diminished appearance on T1WI relative to that reported for older patients, which might indicate a thus far unexplored intrinsic difference in PTPR tumors dependent on the patient’s age.

The clinical behavior, prognosis, and appropriate treatment of PTPR in pediatric patients are yet to be fully defined due to the rarity of these tumors and the limited number of reported cases in the literature. PTPR in adults has a high recurrence rate of up to 67–73%, with a reported 5-year progression-free survival rate of only 27%, while a lower recurrence rate of 47% is reported in children [3].

Surgery is the primary therapy for PTPR, and the extent of surgical resection is the only clinical factor significantly associated with better overall survival. Incomplete surgical resection and tumors with higher mitotic and proliferative activity (as measured by Ki-67 expression) are associated with a poor prognosis [10]. Focal adjuvant radiotherapy plays an important role in controlling subsequent tumor recurrence.

There is no proven benefit of chemotherapy, and different chemotherapy regimens are reported in recurrent refractory cases, including procarbazine, lomustine, vincristine, and temozolomide [11].

At the molecular signaling level, mTOR kinase inhibitors, such as everolimus, are reported to be effective in controlling recurrent PTPR tumors with chromosome 10 deletions and specifically with inactivating mutations in the PTEN (phosphatase and tensin-like protein) tumor suppressor gene on chromosome 10, which is involved in the PI3K/Akt/mTOR signaling pathway. PTEN and its downstream targets may respond to everolimus with or without temozolomide [12].

Based on the DNA methylation patterns, we could discriminate two separate clinical behaviors. First case: The DNA methylation class was PTPR Group B. The patient presented with spinal leptomeningeal dissemination, which is rarely reported in PTPR. Tumor control with CSI radiation therapy alone was excellent, with further tumor volume reduction continuing over time posttreatment.

The second case: The DNA methylation class was PTPR Group A, but the patient had an aggressive course with recurrent local tumor growth, despite radiation therapy and repeated surgical resections without spinal seeding metastasis. Interestingly, this patient responded well to pineoblastoma-like intensive chemotherapy, indicating that there are instances where chemotherapy is indicated in the treatment of PTPR.

The third patient: The DNA methylation class was PTPR Group B. The patient also had local recurrence with microscopic CSF spinal dissemination, and was treated with CSI radiation therapy.

Currently, the clinical behavior of PTPRs can be compared to that of ependymoma, with gross total resection as a mainstay of treatment, followed by surveillance neuroimaging. Adjuvant therapies, such as radiation with or without chemotherapy, should be considered when complete surgical resection is not feasible.

Based on our case series, we recommend using the DNA methylation patterns to detect PTPR Group B, which is reported to have a more aggressive course and was present in our patients with spinal metastasis that needs to be controlled with CSI radiation therapy.

Future therapeutic strategies will undoubtedly benefit from further molecular and mechanistic insights into the effects of the chromosomal changes associated with individual PTPR tumors. This might open the door to individualized medicine approaches that allow therapeutic targeting of specific signaling pathways or molecular mechanisms that play critical roles in the development of an individual’s PTPR tumor.

## Data Availability

Data can be requested from the corresponding author.

## Abbreviations

Papillary tumors of the pineal region (PTPR); King Fahad Medical City (KFMC); Sub commissural organ (SCO); Ventriculoperitoneal shunt (VPS); External beam radiation therapy (EBRT); Intracranial pressure (ICP); Saudi Arabian Pediatric Hematology Oncology Society (SAPHOS).
